# A Transgenic Model for Conditional Induction and Rescue of Portal Hypertension Reveals a Role of VEGF-Mediated Regulation of Sinusoidal Fenestrations

**DOI:** 10.1371/journal.pone.0021478

**Published:** 2011-07-11

**Authors:** Dalit May, Valentin Djonov, Gideon Zamir, Miklosh Bala, Rifaat Safadi, Miriam Sklair-Levy, Eli Keshet

**Affiliations:** 1 Department of Molecular Biology, The Hebrew University–Hadassah University Hospital, Jerusalem, Israel; 2 Institute of Anatomy, University of Berne, Berne, Switzerland; 3 Department of Surgery, The Hebrew University–Hadassah University Hospital, Jerusalem, Israel; 4 Department of Medicine, The Hebrew University–Hadassah University Hospital, Jerusalem, Israel; 5 Department of Radiology, The Hebrew University–Hadassah University Hospital, Jerusalem, Israel; University of Bristol, United Kingdom

## Abstract

Portal hypertension (PH) is a common complication and a leading cause of death in patients with chronic liver diseases. PH is underlined by structural and functional derangement of liver sinusoid vessels and its fenestrated endothelium. Because in most clinical settings PH is accompanied by parenchymal injury, it has been difficult to determine the precise role of microvascular perturbations in causing PH. Reasoning that Vascular Endothelial Growth Factor (VEGF) is required to maintain functional integrity of the hepatic microcirculation, we developed a transgenic mouse system for a liver-specific-, reversible VEGF inhibition. The system is based on conditional induction and de-induction of a VEGF decoy receptor that sequesters VEGF and preclude signaling. VEGF blockade results in sinusoidal endothelial cells (SECs) fenestrations closure and in accumulation and transformation of the normally quiescent hepatic stellate cells, i.e. provoking the two processes underlying sinusoidal capillarization. Importantly, sinusoidal capillarization was sufficient to cause PH and its typical sequela, ascites, splenomegaly and venous collateralization without inflicting parenchymal damage or fibrosis. Remarkably, these dramatic phenotypes were fully reversed within few days from lifting-off VEGF blockade and resultant re-opening of SECs' fenestrations. This study not only uncovered an indispensible role for VEGF in maintaining structure and function of mature SECs, but also highlights the vasculo-centric nature of PH pathogenesis. Unprecedented ability to rescue PH and its secondary manifestations via manipulating a single vascular factor may also be harnessed for examining the potential utility of de-capillarization treatment modalities.

## Introduction

Different insults inflicting hepatocyte damage, such as alcohol or acute and chronic viral infections, may eventually lead to cirrhosis and intra-hepatic portal hypertension (PH). Anatomical changes such as fibrotic scar and regenerative nodule formation that result in mechanical compression of the hepatic vasculature have been traditionally implicated as the dominant cause for increased intra-hepatic vascular resistance, the hallmark of sinusoidal-type PH. It is similarly acknowledged, however, that hepatic stellate cells (HSCs) play a pivotal role in this process. A common pathway in PH pathogenesis due to increased intra-hepatic resistance involves activation of HSCs from a quiescent, vitamin A- storing subendothelial cells to myofibroblast-like cells, endowed with a contractile, proinflammatory and fibrogenic properties [1]. Together, HSC-associated anatomical changes contribute to increased mechanical resistance to blood flow, while contractile activity of activated HSCs might contribute to increased hemodynamic pressure [2]. The sinusoidal endothelium is distinguished by openings (fenestrations) that, together with discontinuities in the basement membrane are essential for proper permeability through this unique low resistance/low pressure microvascular network. Accordingly, matrix deposition within the space of Disse and closure of endothelial fenestrations –processes that together underlie sinusoidal capillarization- impede the rapid exchange of solutes between the sinusoidal space and hepatocytes, causing increased resistance to portal blood flow and PH [3]. Thus, while parenchymal damage is considered to be the initial event in PH pathogenesis, its impact on the hepatic microvasculature appears to be the proximal cause of PH and its sequela. Here we examined whether enforced sinusoidal capillarization, not accompanied by parenchymal architectural derangement may lead to PH. To this end, we have developed a unique transgenic mouse model for perturbing the hepatic vasculature in a conditional and reversible manner via manipulations of Vascular Endothelial Growth Factor (VEGF).

VEGF, in addition to its activity as an angiogenic factor, also thought to play multiple roles in adult vasculatures. Notably, VEGF was shown to induce endothelial fenestration *in vitro* [4], [5]. Likewise, VEGF blockade during development resulted in generation of sinusoidal endothelial cells (SECs) with fewer fenestrations [6]. Other studies have shown that fenestrated endothelium, in general, is more vulnerable to VEGF withdrawal than non-fenestrated endothelium [7]. The notion that ongoing VEGF signaling might be required to maintain fenestrations in adult SECs *in vivo*, however, has not been examined. Here we show that VEGF is indeed essential to maintain SEC fenestrations and HSC quiescence and that, correspondingly, mere blockade of endogenous VEGF is sufficient to cause sinusoidal capillarization which, in turn, leads to PH and its sequela, including ascites, splenomegaly and the development of venuous collateral circulation. Thus, the study highlights the vasculo-centric nature of PH causation and provides a convenient experimental platform to explore PH development without the confounding factors associated with parenchymal damage and independently of its different etiologies.

While rescuing PH and its sequela remains a prime clinical goal, recent animal and clinical works have provided only limited evidence showing that targeting endothelial dysfunction (e.g. by statins) can decrease intra-hepatic vascular resistance of liver vasculature in the cirrhotic liver, reduce portal pressure and result in improved liver function [8].

Here we show that re-opening of SEC fenestrations via restoration of VEGF function fully reverses PH and its secondary manifestations. These finding suggest the deranged sinusoidal network as a possible therapeutic target and attempted de-capillarization as a possible treatment modality.

## Materials and Methods

### Transgenic mice and conditional modulations of VEGF signaling

All animal procedures were approved by the animal care and use committee of the Hebrew University, Approval # MD11904-2 entitled: “Regulation and role of sFlt in physiology and pathophysiology”. A bi-transgenic system for organ specific-, tetracycline-regulated transgene expression was used. Liver-specific induction was obtained by using a driver line in which tTA expression is driven by a C/EBPβ (CCAAT/enhancer binding protein β) promoter (also known as liver-activator protein or PLAP; [9]). The responder tet-soluble VEGF-R1 transgenic line encodes a tetracycline-inducible protein composed of an IgG1-Fc tail fused to the extracellular domain of VEGF-R1 (corresponding to amino acid residues 1 to 631 of human VEGF-R1 containing the ligand binding domain but lacking the trans-membrane and cytoplasmic domains). Induction of soluble VEGF-R1 (sVEGF-R1) in double-transgenic mice by tetracycline withdrawal and its shut-off by tetracycline addition (0.5mg/ml tetracycline and 3% sucrose in the drinking water) were carried-out as previously described [10]. Levels of circulating sVEGF-R1 were determined using a soluble VEGF-R1 ELISA kit (human VEGF R1 Quantikine kits DVR100, R&D systems).

### RNA analysis and *in situ* hybridization

Northern blotting of whole organ RNA was performed as previously described [11]. hVEGF-R1 probe was generated by digesting the expression vector with *Hin*dIII + *Sac*I (1.2kb fragment).

### Histochemistry and Immunohistochemistry

5 um paraffin sections were used for light microscopy; trichrome stain was performed according to Masson Goldner. For vWF (factor VIII) immunestaining – factor VIII rabbit anti-human (DAKO 1∶200) was used.

### Western blot

α-Smooth muscle actin (αSMA) - Whole liver protein extracts were prepared. Blots were incubated with anti-SMA (DAKO, cat no. M0851) mouse monoclonal antibody (1∶2000).

### Transmission electron microscopy

For EM studies, anesthesized mice were perfusion-fixed using a solution of cold 2.5% glutaraldehyde in PBS, post-fixed in osmium tetroxide, block-stained using uranyl acetate, dehydrated through ascending concentrations of ethanol, and embedded in epoxy resin [12]. Ultrathin sections were obtained at 90 nm, counterstained with lead citrate and viewed on a Philips EM-300 microscope.

### Ultrasonography

Abdominal ultrasound was performed on anesthetized mice (100 µl of 17% ketamine, 3% xylazine in 0.9% NaCl, intra-peritoneal) with a L12-15 MHz linear transducer on ATL HDI 5000 (Philips) machine for the presence of ascites.

## Results

### A transgenic system for conditional and reversible suppression of VEGF signaling in the adult liver

**Figure 1:**
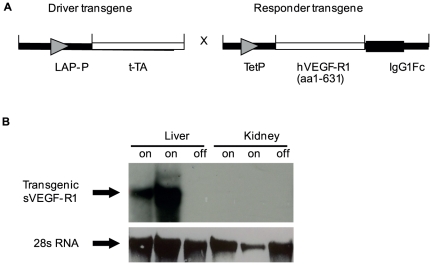
A bi-transgenic system for conditional and reversible suppression of VEGF signaling in the liver. (a) A schematic representation of the driver and responder transgenes used in the bi-transgenic system (b) Northern blot analysis of hepatic and renal RNA with a probe specific for transgenic (human) VEGF-R1. The term “off” indicates control littermates; “on” indicates that sVEGF-R1 expression was induced for 1 month in two transgenic animals. Note the tissue specific expression of the transgene.

In light of evidence that VEGF is constitutively expressed in the adult liver and continuously transmitting VEGF-induced signals [13], we reasoned that VEGF might play a maintenance role in the hepatic circulation. To determine this, we developed a transgenic system allowing for conditional suppression of endogenous VEGF signaling, specifically in the adult liver. The system is based on conditional induction of a secreted soluble VEGF decoy receptor 1 (sVEGF-R1) that sequesters VEGF and precludes its signaling [10]. Briefly, a transgenic line harboring a sVEGF-R1-encoding transgene driven by a tetracycline-responsive promoter (the ‘responder’ line) was mated with a transgenic line harboring a tetracycline-regulated transactivator protein (tTA) driven by the liver-specific C/EBPβ/PLAP promoter [9] (the ‘driver’ line) (Fig. 1). Double transgenic mice were selected for VEGF modulations, whereas littermates that inherited only one of the two transgenes served as controls. The onset of sVEGF-R1 activation in double transgenic animals and the duration of its expression were tightly controlled by including or omitting tetracycline from the drinking water (‘off’ and ‘on’ modes, respectively), which also allowed to restore VEGF function at will. sVEGF-R1 expression was induced in adult mice (over 6 weeks of age) for the indicated time.

A successful ‘on’ switching of sVEGF-R1 was secured by induction of transgenic sVEGF-R1 mRNA in the liver but not in any other organ (Fig. 1) and by detecting the encoded protein in the liver and circulation of double transgenics, mounting to average of 87.4 ng/ml of circulating sVEGF-R1 compared to undetectable levels in control mice. Noteworthy, although sVEGF-R1 produced by the liver was also accessible to the circulation, no phenotype was detected in any organ besides the liver. Conversely, when the soluble VEGF receptor was induced in the heart (using driver lines harboring the cardiac-specific promoter) clear vascular phenotypes were detected exclusively in the heart but not in the liver [10]. These findings indicated that the system is indeed suitable for blocking VEGF signaling in a tissue-specific manner.

Continuous VEGF signaling is required to maintain fenestrations of liver sinusoidal vessels.

**Figure 2:**
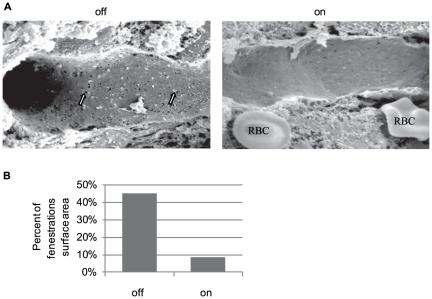
sVEGF-R1 expression in the adult liver causes closure of sinusoidal fenestrations. (a) Scanning electron microscopy of sinusoids in control (‘off’) showing fenestrations arranged in sieve plates (white arrows) and loss of fenestration following one month of switch ‘on’ (sVEGF-R1 expression). RBC = Red Blood Cell (b) Quantification of the percent of fenestrations' surface area in the sinusoids. sinusoidal area. ‘off’−40.5%, ‘on’−8.5%.

High resolution scanning electron microscopy (SEM) was used to reveal ultra-structural changes in SECs subjected to VEGF blockade. Control mice exhibited a typical SEC phenotype distinguished by extensive clusters of fenestrations occupying more than 40% of the endothelial surface area [14]. In contrast, mice subjected to VEGF blockade for 4 weeks exhibited a remarkable 5-fold reduction in fenestration, now occupying only 8.5% of the total surface area (Fig. 2). These results indicated that ongoing VEGF signaling is required for maintaining SEC fenestration and that a loss of VEGF function is sufficient to cause their closure.

### VEGF blockade leads to activation and transformation of hepatic stellate cells (HSCs)

**Figure 3:**
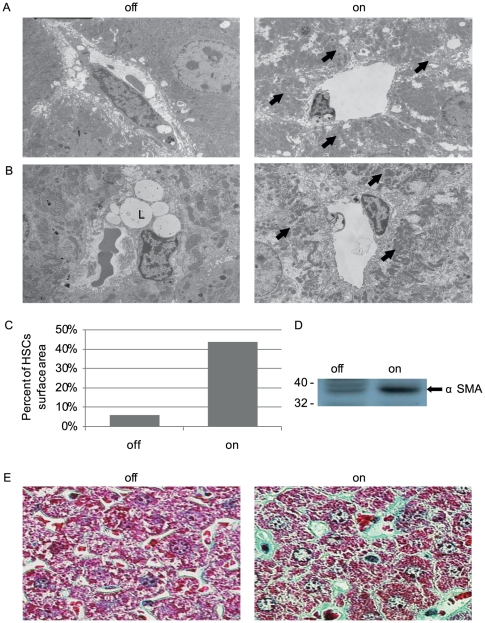
sVEGF-R1 expression in the adult liver causes activation of HSCs. (a) Scanning electron microscopy of sinusoids in ‘off’ (a control littermate) vs. switch ‘on’ liver for one month showing prominent HSCs surrounding sinusoids (arrows) (b) Scanning electron microscopy showing transformation of HSCs from lipid droplets (L) containing cells (‘off’) to myofibroblasts like cells (arrows). (c) Quantification of the surface area of HSCs. ‘off’-5.8%, ‘on’ (sVEGF-R1 expression for one month)-43.7%. (d) Western blot analysis with anti α-smooth muscle actin antibody performed on liver extracts (e) Goldner staining for collagen fibers (green) indicating perisinusoidal accumulation of extra-cellular matrix.

HSCs are known to undergo multiple changes in associating with PH developing on backgrounds of viral- and alcoholic hepatitis [1]. We therefore determined, whether similar HSC changes may take place downstream of VEGF blockade. Results showed that induction of the VEGF-trapping protein indeed led to activation and transformation of HSCs. Indicative of induced HSC accumulation, there was a marked increase in the number of HSCs occupying the peri-sinusoidal space, quantified as a nearly 8-fold increase in the surface area covered by HSCs (Figs. 3A and C). Indicative of HSC transformation were the following observations: I. An ultrastructural change from a lipid-storing cell to a cell devoid of lipid droplets with a myofibroblistic-like cell morphology (Fig. 1A). II. Upregulated expression of α-smooth muscle actin (αSMA) which is normally expressed only by the smooth muscle cells of the larger blood vessels (Fig. 3D). III. Accumulation of perisinusoidal extracellular matrix, shown here by a specific staining for collagen, known to be mainly produced by activated-fibrogenic HSCs (Fig. 3E). Noteworthy, all of the above changes typify HSC changes encountered during the clinical course of PH and considered to be critical for its pathogenesis.

### Inhibition of VEGF in the liver causes sinusoidal capillarization

**Figure 4:**
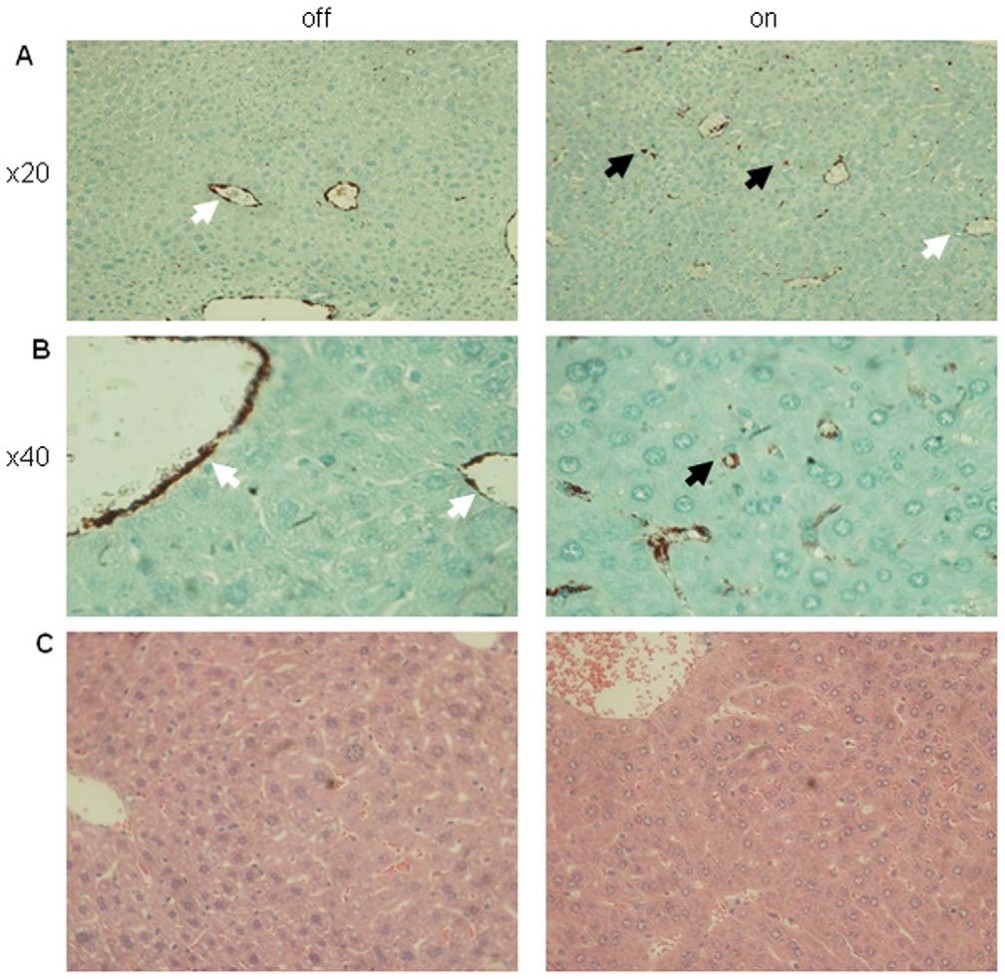
Sinusoidal capillarization in sVEGF-R1 expressing livers without parenchymal damage. (a,b) Immunohistochemical staining for vWF (Von-Willebrand Factor) on liver sections (black arrows-sinusoids, white arrows-larger blood vessels). (c) H&E staining of liver sections showing normal appearance.

Overall, the data indicated that loss of VEGF function results in sinusoidal capillarization, i.e. the combined loss of SEC fenestrations and deposition of basement membrane by activated HSC [3]. This process of sinusoidal de-differentiation is associated with marked changes in the repertoire of endothelial-expressed genes, notably acquisition of von-Willebrand factor (vWF) expression, a protein not normally expressed by SECs and upregulated during liver damage [15], [16], [17] As shown in fig. 4, sinusoidal capillarization induced by VEGF blockade was also manifested by sinusoidal expression of vWF. Thus, ongoing VEGF signaling appears essential for maintaining the differentiated state of liver sinusoids.

Sinusoidal capillarization was not associated with any detectable parenchymal damage or bridging fibrosis, as evidenced by the normal morphology of hepatocytes and by failure to detect a regenerative liver response, bile duct pathology (Fig. 4 C), as well as hepatocyte cell death (not shown).

### VEGF inhibition culminates in portal hypertension and its typical complications

**Figure 5:**
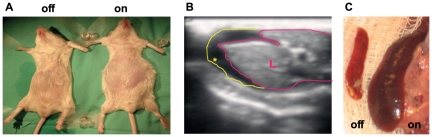
Complications of portal hypertension resulting from sVEGF-R1 expression in the liver. (a) Anesthetized mice showing abdominal distension. ‘on’-sVEGF-R1 expression for one month. (b) Abdominal ultrasonography documenting ascites after one month of sVEGF-R1 expression in the liver. L-liver lined with pink line, ascites (hypoechogenic) is marked by a yellow asterisk and a yellow line. (c) Representative spleens taken from control (‘off’) and after one month of sVEGF-R1 expression (‘on’) in the liver. Average spleen weight is 100 mg and 193 mg in control and transgenic mice, respectively.

Next, we wished to determine whether sinusoidal capillarization caused by VEGF withdrawal might also produce all the sequela of PH despite the otherwise normal liver morphology [2], [18].To this end, we examined mice at one month after the onset of sVEGF-R1 induction, focusing on extra-hepatic phenotypes. The development of prominent ascites was clearly evident by abdominal swelling (Fig. 5A) and corroborated by ultrasonography (Fig. 5B). Splenomegaly was evident by a twofold enlargement and congestion of the spleen (Fig. 5C). A later compensatory response to PH often observed in the clinical setting, namely the formation of mesenteric venous collaterals, was also evident (not shown). Thus, the liver pathology induced by VEGF inhibition fully reproduces the typical clinical sequela of PH.

### Portal hypertension is reversible upon VEGF reactivation

In light of current debate to what extent can PH-associated fibrosis and secondary complication be reversed upon terminating the initial cause of injury, we wished to determine whether intra- and extra-hepatic abnormalities in our experimental system can be resolved upon restoring VEGF function. To this end, mice maintained for one month in the sVEGF-R1 ‘on’ mode, i.e. at a time point where all of the above symptoms has already been produced (and secured by observing abdominal ascites by ultrasonography) were transferred to the switch ‘off’ conditions to re-gain VEGF function (‘on→off’ mode). Noteworthy, when the ‘on’ switch was kept for a longer time, further deterioration resulted in animal mortality.

**Figure 6:**
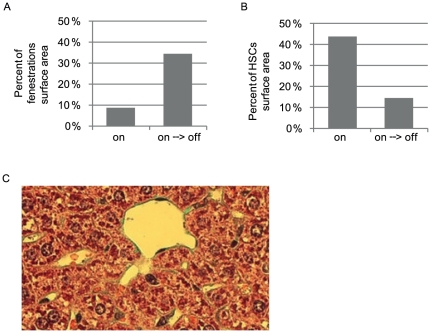
Liver phenotype is reversible upon relieving sVEGF-R1. One week after sVEGF-R1 shut off (on→off) (a) Quantification of the percent of fenestrations' surface area in the sinusoids. ‘on’−8.5%, ‘on→off’−34.4% (b)-quantification of area covered by HSCs ‘on’−43.7%, ‘on→off’ (‘on’ for one month then ‘off’ for one week)−14.4% (c) Goldner staining highlighting collagen fibers in green showing reduced extracellular-matrix deposition perisinusoidally compared to ‘on’ (fig 3E).

Remarkably, all intra- and extra-hepatic phenotypes were reversed with within 1 week of VEGF function restoration and mice became indistinguishable from controls (due to the longer half-life of collagen, its reduction was apparent with a few weeks delay). This was evident as follows: SEC fenestrations re-appeared (Fig. 6A, to be compared with the onwards switch in fig. 2A). HSCs returned to a quiescent state as manifested in normal abundance in the peri-sinusoidal space (Fig. 6B, compared with the onward switch in Fig. 3C). The amount of peri-sinusoidal collagen was reduced to an apparently close to normal level (Fig. 6C compared to Fig. 3E). As anticipated from the re-gain of normal sinusoidal structure and presumed return to a low resistance state, secondary complications also disappeared including resolution of ascites and venous collaterals and regression of splenomegaly (not shown).

## Discussion

Here we harnessed a conditional transgenic system for liver-specific modulations of VEGF function to show that VEGF blockade leads to closure of SEC fenestration and to activation of peri-sinusoidal HSCs, acting in concert to induce sinusoidal capillarization. Enforced capillarization was sufficient to produce significant PH and its secondary complications in the absence of a detectable parenchymal damage. These results point out that liver fibrosis is not a mandatory requirement for PH development and highlight the key role of vascular perturbation as the proximal cause of PH. To our knowledge this is the first example of a PH animal model where loss-of-function of a single protein is sufficient to produce all hallmarks of the condition.

Initially shown to function as an angiogenic factor, VEGF was subsequently shown to play different roles in the maintenance of adult vasculatures. Noteworthy, VEGF is suggested to play a role in control of systemic blood pressure, as evidenced by induced hypertension in patients treated with the VEGF-neutralizing antibody Bevacizumab [19]. Here, we uncovered yet another function of VEGF, namely, a requirement for ongoing VEGF signaling in order to keep SECs' fenestrations in an open state. This was supported by closure of fenestrae upon inhibition of endogenous VEGF function and their re-opening upon restoration of VEGF function. We note that while ability of VEGF to form new fenestrae has been previously documented [13], [20], a requirement for VEGF to maintain already formed fenestrae in mature SECs *in vivo* was not previously shown, as it necessitated the use of an on/off genetic switch system. These results are supported by a clinical trial findings that removing VEGF from the hepatic microvasculature in the setting of cirrhosis and portal hypertension has deletarious effect [21].

The conditional VEGF switch system employed in this study provides several advantages over previously used methodologies of *in vivo* VEGF modulations. First, VEGF blockade takes place only in the relevant organ, thus circumventing systemic influences. We note in this regard that although the induced decoy receptor is a secreted protein, its peri-cellular retention is the likely explanation to our cumulative experience that vascular phenotypes are solely observed in the particular organ where it is induced. Second, unlike other methodologies of VEGF loss of function, which are often incomplete, this system allows to attain complete VEGF blockade. Third, the option to induce and to terminate the VEGF blockade at any given schedule is instrumental for examining adult phenotypes and their reversal.

The finding that VEGF is required to maintain SEC fenestrations, in conjunction with the fact that fenestrations are of essence for maintaining a permeable, low-resistance portal circulation, provides an explanation to the finding that enforced VEGF blockade results in increased resistance and portal hypertension.

It should be pointed-out, however, that there is no evidence that a loss of VEGF function is an etiological factor in clinical settings of PH. Nevertheless, this experimental model might be useful for dissecting the overall pathogenic process to its individual contributing sub-process. This is exemplified here by singling-out sinusoidal capillarization as the key contributor to PH development.

Moreover, this model more closely resembles disorders distinguished by a primary damage to the liver vasculature, such as Budd-Chiari syndrome and Hepatic veno-occlusive disease (hepatic sinusoidal obstruction syndrome), which are both characterized by hepatic venous outflow obstruction at different levels (for review see [22]), causing hepatic sinusoidal hydrostatic pressure elevation and portal hypertension with variable stages of parenchymal damage.

VEGF suppression in the adult liver also leads to activation of HSCs from quiescence into a proliferative state and their transformation from retinol-storing cells to α-smooth muscle actin-expressing myofibroblast-like cells engaged in deposition of extracellular matrix proteins in the space of Disse. Interestingly, a previous study reported that upregulation of VEGF activates HSCs [23], whereas other studies have shown that addition of recombinant VEGF to the culture medium attenuates an increase in α-smooth muscle actin expression in these cells, suggesting that VEGF is indeed required for the maintenance of the differentiated state of HSCs [24], [25]. We note, however, that our study is an *in vivo* study aimed at measuring maintence functions of VEGF in the normal liver and also differ in this respect from *in vivo* studies examining the effects of VEGF on HSCs in a pathological cirrhosis model. Currently it is not known whether VEGF induces these HSC changes directly or indirectly. An indirect effect was suggested in an *in vivo* study, showing that HSC quiescence requires both VEGF and co-culture with SECs. Therefore, VEGF sequesetration could lead to closure of fenestration which, in turn, is likely to result in hypoxia. Hypoxia-inducible factors other than VEGF, such as PDGF could then activate HSCs [23], [24], [25], [26].

The study shows that PH, as well as its secondary complications, ascites, splenomegaly and formation of venous collaterals can be fully reversed by VEGF reactivation. Because the natural proximal causes of sinusoidal capillarization are unknown and unless it is caused by a loss of VEGF function, enforcing de-capilarization for clinical purposes requires further mechanistic insights. Nevertheless, our experimental system might lend itself as suitable experimental platform in examining potential drugs with respect to the feasibility of reversing sinusoidal capillarization and thereby rescue PH and its complications.
